# Serum HBV pregenomic RNA exhibited opposite associations with NK^dim^ and NK^bright^ cell immunity in treatment-naïve chronic hepatitis B patients

**DOI:** 10.1042/BSR20210600

**Published:** 2021-07-02

**Authors:** Yurong Gu, Zexuan Huang, Xiaoyan Li, Youming Chen, Chunhong Liao, Yanhua Bi, Yuehua Huang

**Affiliations:** 1 Department of Infectious Diseases, The Third Affiliated Hospital of Sun Yat-sen University, Guangzhou, China; 2 Guangdong Provincial Key Laboratory of Liver Disease Research, The Third Affiliated Hospital of Sun Yat-sen University, Guangzhou, China

**Keywords:** hepatitis B virus (HBV), natural killer (NK) cells, pregenomic RNA (pgRNA)

## Abstract

Hepatitis B virus (HBV) pregenomic RNA (pgRNA) is a new biomarker that reflects HBV replication, but its relationship with natural killer (NK) cell immunity in chronic hepatitis B (CHB) is unknown. We assessed serum HBV pgRNA levels in 323 CHB patients by reverse transcription-polymerase chain reaction, assessed cytokine production and activation and inhibitory markers of NK cells by flow cytometry, and measured serum cytokines by enzyme-linked immunosorbent assays (ELISAs). Among the different CHB phases, the serum HBV pgRNA level was highest in the immune-tolerant (IT) and immune-active (IA) phases. Regarding NK and NK^dim^ cells, HBV pgRNA was negatively associated with frequencies, but positively associated with NKp44 and NKp46 expression (activation markers). Regarding NK^bright^ cells, serum HBV pgRNA was positively associated with frequency and programmed cell death protein 1 (PD1) expression (inhibitory marker), but negatively associated with NKp44 and NKp46. Serum HBV pgRNA was not associated with NKp30 (activation marker) on NK cells or subsets. Lastly, serum HBV pgRNA was positively correlated with the levels of serum IL-7 and IL-12P40 (NK cell-promoting cytokines) and negatively correlated with serum prostaglandin E2 (PGE2) level (which negatively regulates NK cells). In conclusion, we found varied relationships between serum HBV pgRNA and NK cells and subsets, indicating that HBV pgRNA may play a complicated role in NK cell-related immunity, providing new information on HBV and host immunity.

## Introduction

Hepatitis B virus (HBV) is one of the most common viruses that can infect the liver, especially in Asia. Chronic HBV infection can cause prolonged liver injury, which may progress to liver cirrhosis, hepatic failure, and even hepatocellular carcinoma [1]. HBV is a non-cytopathic virus and its mechanism of liver injury is thought to involve inducing immune inflammation [2].

Natural killer (NK) cells belong to the innate immune system and play crucial antitumor and antivirus roles. They directly target cells, causing apoptosis or osmotic cell lysis via cytotoxic mechanisms [3]. Based on the expression of surface marker CD56, NK cells can be subtyped into CD56^bright^ (NK^bright^) cells with high expression of CD56 and CD56^dim^ (NK^dim^) cells with low expression of CD56. The majority of NK cells in peripheral blood are NK^dim^ cells with typical cytotoxicity and low cytokine expression. In contrast, NK^bright^ cells, which are usually non-cytotoxic and express high levels of cytokines, are significantly increased in inflammatory sites [4].

NK cells have an important and early role in HBV infection. In chimpanzee models of acute HBV infection, HBV DNA is significantly reduced by non-T cells, especially NK cells, long before liver infiltration of T cells and liver injury, indicating that NK cells contribute to HBV clearance [5]. Liver infiltration of NK cells and identification of infected cells without major histocompatibility complex (MHC) I expression reflect the early antiviral function of NK cells [6]. NK cells may be able to act via non-cell solution mechanisms, rather than by lysing liver cells, which could help to avoid liver damage during acute HBV infection [7]. However, during chronic HBV infection, circulating NK cells have dysfunctional antiviral capacities regarding cell activation, cytokine production, and cytotoxicity [8–10].

The factors that cause NK cell impairment during chronic HBV infection are very complex. High HBV loads and antigens, such as HBV e antigen (HBeAg), HBV surface antigen (HBsAg) and HBV DNA, can affect innate and adaptive immunity in chronic hepatitis B (CHB) patients. Sustained high levels of viral antigens can induce T-cell dysfunction by direct action against T cells or indirect suppression of cytokine regulation [11]. Additionally, HBV can cause dysfunction of innate immune cells, including NK cells. Yang et al. found that HBV antigen could paralyze NK cell immunity by suppressing the nuclear factor (NF)-κB, signal transducer and activator of transcription 1 (STAT1), and mitogen-activated protein kinase (MAPK) signaling pathways, causing an imbalance between NK cell activation and inhibition [12,13]. Furthermore, instead of clearing the virus, as they do in acute HBV infection, NK cells promoted liver injury by inducing cytotoxicity in chronic HBV infection. Additionally, CHB patients with sustained high HBsAg levels after nucleos(t)ide analog cessation exhibited a flare of liver inflammation due to increased circulating NK cells [14].

Recently, HBV pregenomic RNA (HBV pgRNA) has emerged as a biomarker of HBV replication. It is transcribed from HBV covalently closed circular DNA (cccDNA), the template for HBV RNA transcription, in the nucleus of infected liver cells. In our previous study [15], we found that serum HBV pgRNA was associated with T-cell immunity, suppressing Th1 immunity but promoting Th2 immunity. However, studies have rarely focused on HBV pgRNA and NK cell immunity in chronic HBV infection. In the present study, we assessed serum HBV pgRNA and NK cell immune characteristics in 323 antiviral therapy-naïve CHB patients, and we explored the associations between them.

## Materials and methods

### Patients

Patients with chronic HBV infection who had never received antiviral therapy were recruited from the Liver Clinic of the Third Affiliated Hospital of Sun Yat-sen University. Patients with hepatitis C virus (HCV), hepatitis D virus, or human immunodeficiency virus (HIV) co-infection, patients with cirrhosis, liver carcinoma or autoimmune disorders, and patients receiving immunosuppressive therapy were excluded. All of the patients signed written informed consent forms. The study was approved by the Ethics Committee of the hospital and conformed to the Declaration of Helsinki.

The 323 eligible CHB patients were classified into the following four groups based on international CHB treatment guidelines related to virological and biochemical parameters (Table 1): immune tolerant (IT), immune active (IA), inactive carriers (ICs), and gray zone (GZ) groups [16]. Sixteen healthy controls (HCs) were included in the study. The demographic and clinical-virological characteristics of participants are listed in Table 2, including HBV DNA load, HBV antigens and antibodies, hepatic panel results (alanine aminotransferase [ALT], aspartate aminotransferase [AST], albumin [ALB] total bilirubin [TBIL]), and liver fibrosis result (FibroScan value).

**Table 1:** Disease phase classification criteria

Classification	ALT	HBV DNA	HBeAg
IT	Normal	>1 million IU/ml	Positive
IA	Elevated	>20000 IU/ml	Positive
		>2000 IU/ml	Negative
Inactive CHB (IC)	Normal	Low HBV DNA level	Negative
GZ	Not classified as IC, IT, or IA		

Upper limit of normal (ULN) of ALT: 30 U/l for males and 19 U/l for females. The table was cited from the American Association for the Study of Liver Disease guidelines, 2018

**Table 2:** Clinical-virological characteristics of CHB patients included in the study

Characteristics	IT (*n*=29)	IA (*n*=190)	IC (*n*=48)	GZ (*n*=56)	HC (*n*=16)	*P*-value
Age, years, median (quartile)	27.1 (24, 31)	30.9 (25, 35)	33.13 (27.25, 38.5)	32.52 (26.25, 38)	27 (25, 44)	***0.003***
Sex						0.407
Male, *n* (%)	18 (61.3%)	145 (76.3%)	34 (70.8%)	41 (73.2%)	11	
Female, *n* (%)	11 (38.7%)	45 (23.7%)	14 (29.2%)	15 (26.8%)	5	
BMI, median (quartile)	20.94 (18.5, 22.97)	21.46 (19.26, 22.89)	22.14 (20.20, 23.39)	21.57 (19.54, 23.37)	20.8 (19.1, 24.0)	0.347
AST, U/l, median (quartile)	25.14 (19.5, 29)	110.9 (36, 108.3)	25 (21, 29)	28.3 (22, 31)	22.5 (19.75, 24.75)	***0.000***
ALT, U/l, median (quartile)	24.07 (19.5, 29)	164.3 (55, 175)	22.67 (16.25, 29)	31.14 (20, 32.75)	16.0 (13.2, 19.0)	***0.000***
ALB, g/l, median (quartile)	45.46 (44, 47.25)	44.5 (42.5, 47.1)	46.71 (45.1, 48.45)	46.43 (45.45, 48.03)	45.7 (44.2, 48.0)	***0.000***
GLB, g/l, median (quartile)	28.67 (26.18, 31.47)	29.25 (26.17, 32.21)	28.86 (26.97, 31.13)	29.10 (26.73, 31.39)	29.37 (26.07, 32.26)	0.886
TBIL, μmol/l, median (quartile)	13.56 (9.28, 17.8)	21.25 (28.74, 2.107)	13.22 (9.38, 16.65)	13.32 (9.15, 15.6)	9.0 (8.4, 11.8)	***0.034***
Fibroscan, Kpa, median (quartile)	4.8 (4, 5.63)	9.46 (5.5, 10.4)	5.15 (4.23, 5.58)	5.16 (4.15, 5.5)	4.5 (4.0, 5.0)	***0.000***
HBeAg status						***0.000***
Negative, *n* (%)	0 (0%)	42 (22.1%)	45 (93.8%)	48 (85.7%)		
Positive, *n* (%)	29 (100%)	148 (77.9%)	3 (6.3%)	8 (14.3%)		
Log_10_ HBsAg, IU/ml, median (quartile)	4.47 (4.44, 4.72)	3.885 (3.42, 4.49)	2.81 (2.035, 3.56)	2.86 (2.23, 3.42)		***0.000***
Log_10_ HBV DNA, IU/ml, median (quartile)	8.04 (8.23, 8.23)	7.08 (6.26, 8.23)	2.07 (1.41, 3)	3.6 (2.98, 4.48)		***0.000***

Abbreviation: GLB, globulin. *P*<0.05 was significant.

### Clinical and serological parameters

ALT, AST, TBIL, and ALB were measured using a 7600-020 (ISE) automatic Analyzer (4, Tokyo, Japan). HBV DNA load was assessed using the Roche AmpliPrep/COBAS TaqMan test (range: 20 to 1.7E+08 IU/ml; Roche Molecular Diagnostics, Branchburg, NJ). HBsAg, HBsAb, HBeAg, HBeAb, and HBcAb were measured using commercial kits (Abbott Laboratory, North Chicago, IL). The FibroScan value was calculated based on liver stiffness (FibroScan, Echosens, Paris, France).

### Quantification of serum HBV pgRNA

The serum HBV pgRNA level was assessed using an HBV pgRNA Kit (SUPBIO, Guangzhou, China). Briefly, serum HBV RNA was isolated, treated with DNase I, and reverse transcribed according to the manufacturer’s instructions. qPCR was then performed without using reverse transcriptase. The detection limit was 25 copies/ml.

### Cell-surface and intracellular cytokine staining and serum cytokine assays

Peripheral blood mononuclear cells (PBMCs) were isolated using density gradient centrifugation. For cell-surface staining, the PBMCs were incubated with phycoerythrin (PE)-CF594-CD3, fluorescein isothiocyanate (FITC)-CD56, Brilliant Violet 421 (BV421)-programmed cell death protein 1 (PD1), allophycocyanin (APC)-T cell immunoglobulin and mucin domain-containing protein 3 (Tim3), PE-leukocyte-associated immunoglobulin-like receptor 1 (LAIR1), PE-NKp44, PE-Cy7-NKp46, APC-NKG2A, or APC-NKp30 monoclonal antibodies (BD Biosciences) for 30 min at 4°C followed by washing. For intracellular cytokine staining, Leukocyte Activation Cocktail (eBioscience) was used to stimulate the PBMCs at 37°C for 4 h, followed by fixing and permeabilizing the cells using Cytofix/Cytoperm fixation/permeabilization solution (eBioscience). The intracellular cytokines were stained using FITC-interferon (IFN)-γ and PE-Cy7-tumor necrosis factor (TNF)-α monoclonal antibodies (eBioscience). Negative controls (isotype-matched control antibodies; eBioscience and BD Biosciences) were also used. The samples were assessed using a Gallios flow cytometer (Beckman Coulter, Brea, CA) and analyzed using FlowJo software (Ashland, OR).

Serum cytokines, comprising IL-7, IL-12 and prostaglandin E2 (PGE2), were examined using enzyme-linked immunosorbent assay (ELISA) kits (eBioscience, San Diego, CA) according to the manufacturer’s instructions.

### Statistical analysis

Serum HBV pgRNA was expressed as log_10_ copies/ml, and presented as median (lower and upper quartiles). To compare the patient groups, the Wilcoxon signed-rank test was used for continuous variables, Mann–Whitney U test was used for comparison between two groups, and Kruskal–Wallis (K–W) test were for comparison among multiple groups (if the *P-*value of K–W test was significant, then Bonferroni method was used for multiple comparison). The χ^2^ test was used for categorical variables. Correlations were assessed using Spearman correlation analysis. Additionally, the associations of serum HBV pgRNA with various factors were determined by linear regression analyses. All the analyses were performed using SPSS version 23 (IBM Corp., U.S.A.) and *P*<0.05 was considered significant.

## Results

### Associations of serum HBV pgRNA level with clinical-virological characteristics

**Figure 1:**
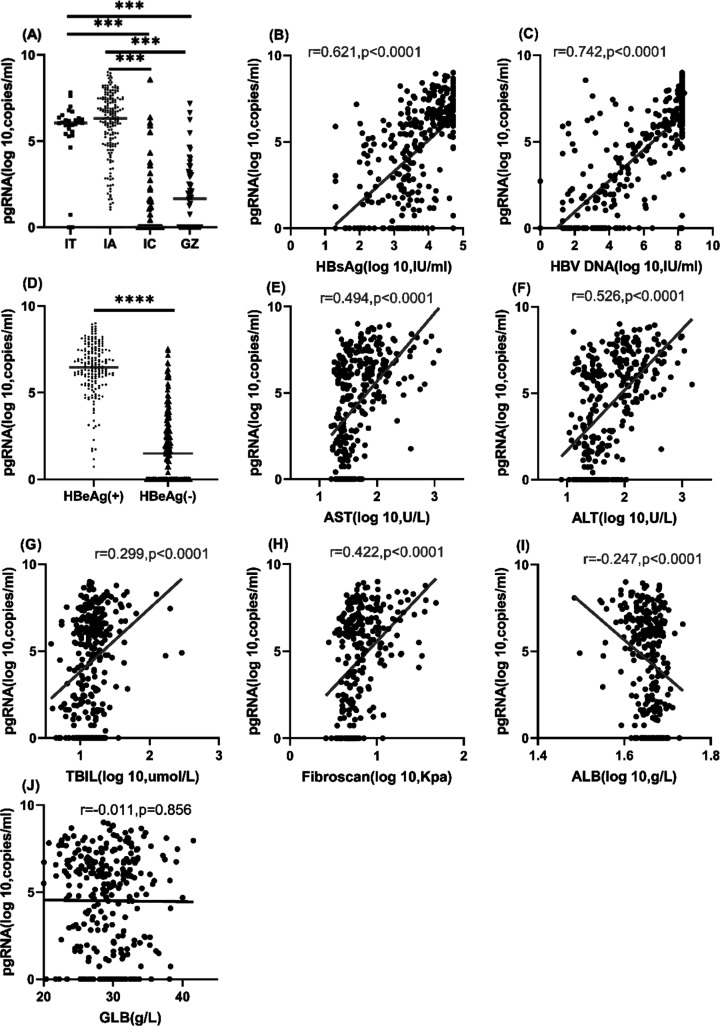
Serum HBV pgRNA level in CHB patients and its relationship with clinic-virological characteristics (**A**) Serum HBV pgRNA level was significantly different among the IT, IA, IC and GZ groups, and it was highest in the IA group. (**B**) Correlation of serum HBV pgRNA with HBsAg levels. (**C**) Correlation of serum HBV pgRNA with HBV DNA levels. (**D**) Serum HBV pgRNA level was higher in HBeAg(+) CHB patients than in HBeAg(−) CHB patients. (**E–J**) Correlations of serum HBV pgRNA with liver inflammation and fibrosis. ***P<0.001, ****P<0.0001.

Based on international guidelines, the patients were divided into IT (*n*=29), IA (*n*=190), IC (*n*=48), and GZ (*n*=56) groups. Demographic and clinical-virological characteristics of the patients and HCs are presented in Table 2. The median serum HBV pgRNA level in the IT, IA, IC, and GZ groups was 5.54 (0, 7.81), 5.71 (0, 9.00), 1.39 (0, 8.55), and 1.76 (0, 7.17) log_10_ copies/ml, respectively. It was significantly higher in the IT and IA groups than the IC and GZ groups using K–W test and Bonferroni method analyses (*P*<0.001) (Figure 1A).

PgRNA originates from HBV cccDNA, which is the template for virus replication. Therefore, we analyzed the relationships between serum HBV pgRNA and virological characteristics. Serum HBV pgRNA was positively correlated with HBsAg level and HBV DNA load (r=0.621 and 0.742, both *P*<0.0001) in the Spearman correlation analysis (Figure 1B,C). Compared with HBeAg(−) patients, HBeAg(+) patients had a significantly higher serum HBV pgRNA level analyzed with Mann–Whitney U test (6.460 [0.000, 9.000] vs 1.490 [0.000, 7.540] log_10_ copies/ml, *P*<0.0001) (Figure 1D). Furthermore, both univariate and multivariate linear regression analyses showed that serum HBV pgRNA was positively associated with HBV DNA load (B =0.903 and 0.564, both *P*<0.001, respectively) and HBeAg (B=4.341 and 2.097, *P*<0.001). Additionally, univariate linear regression showed that serum HBV pgRNA was positively associated with the HBsAg level (B=1.845, *P*<0.001) (Supplementary Table S1).

Although HBV is a non-cytopathic virus, it can cause liver injury indirectly by inducing an immune response. We further explored whether serum HBV pgRNA was associated with liver inflammation and fibrosis. Serum HBV pgRNA was positively correlated with AST, ALT, TBIL, and FibroScan value (r=0.494, 0.526, 0.299 and 0.422, and *P*<0.0001, respectively), negatively correlated with ALB (r=−0.247, *P*<0.0001), and not associated with globulin (GLB) (*P*>0.05) (Figure 1E–J). Both univariate and multivariate linear regression analyses demonstrated that serum HBV pgRNA was positively associated with ALT (B=0.006 and 0.005, *P*<0.001) and FibroScan value (B=0.155 and 0.089, both *P*<0.005). Univariate linear regression analysis showed that serum HBV pgRNA was also positively associated with AST and TBIL (B=0.007 and 0.019, both *P*<0.01), but negatively associated with ALB (B=−0.219, *P*<0.001) (Supplementary Table S1).

Consistent with the distribution of serum HBV pgRNA across the patient groups and its positive correlations with clinical-virological characteristics (Figure 1), the HBsAg level and HBV DNA load were also higher in the IT and IA groups than the IC and GZ groups, and highest in the IT group using the K–W test and Bonferroni method analyses. Liver inflammation (ALT and AST) and fibrosis (FibroScan value) were also highest in the IA group among the four groups. TBIL and ALB were higher in the IT group than the IC and GZ groups. While GLB was not different among the groups (Supplementary Figure S1).

### Associations with NK cell and subset frequencies

The immune system plays important roles in virus clearance and liver injury during chronic HBV infection. In our previous study, we found that serum HBV pgRNA was associated with T helper (Th) cell immunity. Therefore, here we explored the relationship between HBV pgRNA and NK cell immunity.

**Figure 2:**
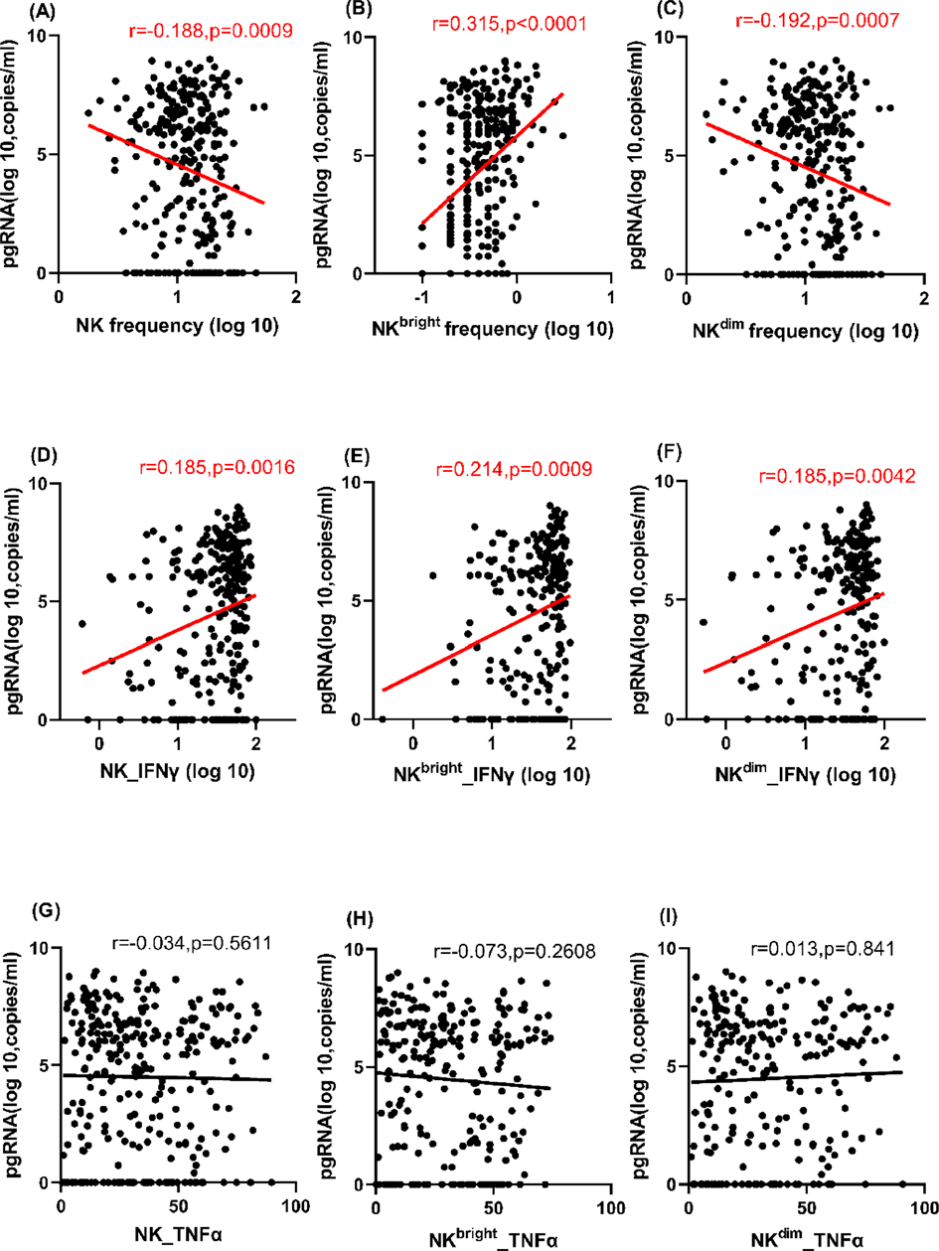
Correlations of serum HBV pgRNA with frequencies and antiviral cytokines produced by NK cells and subsets Correlations of serum HBV pgRNA with (**A–C**) frequencies and (**D–I**) antiviral cytokines (IFN-γ and TNF-α) of NK cells and subsets.

Firstly, the associations of serum HBV pgRNA with NK cell and subset frequencies were analyzed. Serum HBV pgRNA was negatively correlated with NK and NK^dim^ cell frequencies, but positively correlated with NK^bright^ cell frequency (r=−0.188, −0.192 and 0.315, respectively, all *P*<0.001) in the Spearman correlation analysis (Figure 2A–C). Both univariate and multivariate linear regression analyses showed that serum HBV pgRNA was negatively associated with NK cell frequency (B = −0.073 and −0.658, *P*=0.001 and 0.022, respectively) but positively associated with NK^bright^ cell frequency (B=2.489 and 2.885, both *P*<0.001). It was inconsistently associated with NK^dim^ cell frequency based on univariate (B=−0.076, *P*<0.001) and multivariate (B=0.602, *P*=0.039) linear regression analyses (Supplementary Table S2). In summary, serum HBV pgRNA tended to be negatively correlated with NK and NK^dim^ cell frequencies, but positively correlated with NK^bright^ cell frequency.

To gain full understanding of the relationship between serum HBV pgRNA and NK cell immunity, we analyzed the data further using the K–W test and Bonferroni method, assessing the differences in NK cell frequencies among patient groups. NK and NK^dim^ cell frequencies were significantly lower in the IA group than the GZ group, while NK^bright^ cell frequency was higher in the IA group than the IC and GZ groups, which was consistent with the varying correlations between serum HBV pgRNA and NK cell and subset frequencies (Supplementary Figure S2A–C).

### Associations with antiviral cytokines produced by NK cells and subsets

IFN-γ and TNF-α are important antiviral cytokines produced by NK cells in HBV infection, so we explored the associations between serum HBV pgRNA and these cytokines. The results showed that serum HBV pgRNA was positively correlated with IFN-γ produced by NK, NK^bright^, and NK^dim^ cells (r=0.185, 0.214, and 0.185, and *P=*0.0016, 0.0009, and 0.0042, respectively), but there were no correlations with TNF-α produced by NK, NK^bright^, or NK^dim^ cells (*P*>0.05) in the Spearman correlation analysis (Figure 2D–I). Serum HBV pgRNA was positively associated with IFN-γ produced by NK^bright^ cells in both univariate and multivariate linear regression analyses (B=0.025 and 0.035, both *P*<0.005) and IFN-γ produced by NK and NK^dim^ cells in univariate linear regression analysis (B=0.025 and 0.026, *P*=0.001 and 0.002, respectively). Serum HBV pgRNA was positively associated with TNF-α produced by NK cells (B=0.030, *P*=0.017) but negatively associated with TNF-α produced by NK^bright^ cells (B=−0.055, *P*<0.001) in multivariate linear regression analyses (Supplementary Table S2).

Additionally, by the K–W test and Bonferroni method, IFN-γ levels produced by NK cells and subsets were higher in the IA group than the IT and GZ groups and that of NK^bright^ cells was also higher in the IA group than the IC group. The TNF-α level produced by NK cells and subsets did not differ among the groups, which is consistent with the lack of correlation between serum HBV pgRNA and TNF-α level (Supplementary Figure S2D–I).

### Associations with activation markers on NK cells and subsets

**Figure 3:**
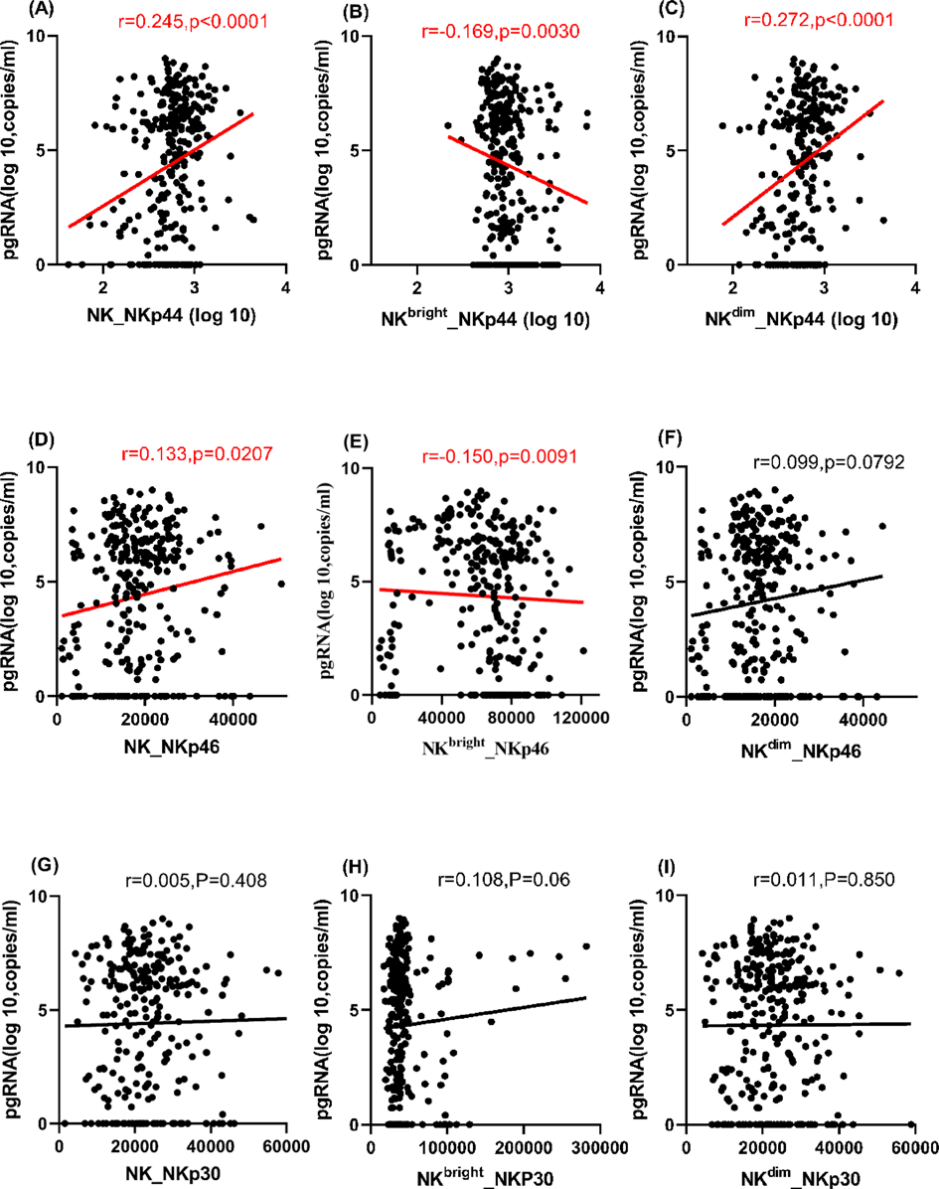
Correlations of serum HBV pgRNA with activation markers (NKP44, NKP46, and NKP30) on NK cells and subsets Correlations of serum HBV pgRNA with (**A–C**) NKP44, (**D–F**) NKP46, and (**G–I**) NKP30 on NK cells and subsets.

NKp44, NKp46, and NKp30 are markers of NK cell activation. The associations of the serum HBV pgRNA level with these markers on NK cells and subsets were analyzed. Serum HBV pgRNA was positively correlated with both NKp44 and NKp46 on NK cells (r=0.245 and 0.133, both *P*<0.05) and NKp44 on NK^dim^ cells (r=0.272, *P*<0.0001), but negatively correlated with NKp44 and NKp46 on NK^bright^ cells (r=−0.169 and −0.150, both *P*<0.01) in the Spearman correlation analysis (Figure 3A–F). Both univariate and multivariate linear regression analyses showed that serum HBV pgRNA was positively associated with NKp44 and NKp46 on NK and NK^dim^ cells (*P*<0.05), except for NKp46 on NK^dim^ cells in univariate linear regression analysis (*P*=0.091). In contrast, it was negatively associated with NKp44 and NKp46 on NK^bright^ cells in multivariate linear regression analysis (B=−0.045 and −0.003, both *P*<0.005). It was not associated with NKp30 on NK cells or subsets in the correlation (Figure 3G–I) or linear regression (*P*>0.05) analyses (Supplementary Table S3). In summary, varying associations were found between serum HBV pgRNA and the activation markers on different NK cells.

In addition, the expression of the activation marker NKp44 on NK and NK^dim^ cells was higher in the IA group than the GZ group, and NKp30 on NK^bright^ cells was lower in IC group than the IA and GZ groups using the K–W test and Bonferroni method analyses. There were no differences in NKp46 on NK cells or subsets among the four groups (Supplementary Figure S3).

### Associations with inhibitory markers on NK cells and subsets

**Figure 4:**
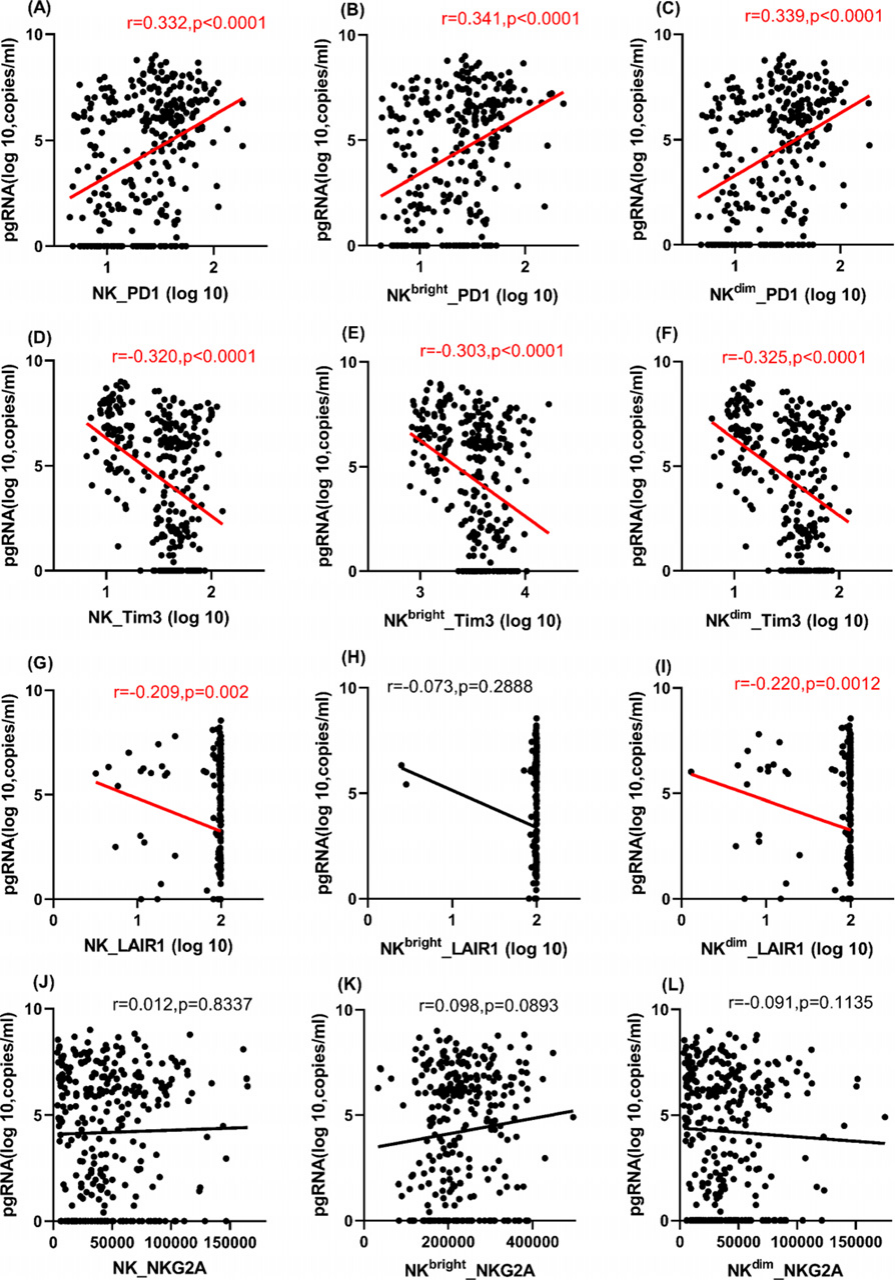
Correlations of serum HBV pgRNA with inhibitory markers (PD1, Tim3, LAIR1, and NKG2A) on NK cells and subsets Correlations of serum HBV pgRNA with (**A–C**) PD1, (**D–F**) Tim3, (**G–I**) LAIR1, and (**J–L**) NKG2A on NK cells and subsets.

NKG2A, PD1, Tim3, and LAIR1 are inhibitory markers of NK cells. The associations of serum HBV pgRNA with these markers on NK cells and subsets were analyzed. Serum HBV pgRNA was positively correlated with PD1 on NK, NK^bright^, and NK^dim^ cells (r=0.332, 0.341 and 0.339, and *P*<0.0001, respectively), but negatively correlated with Tim3 on NK, NK^bright^, and NK^dim^ cells (r=−0.320, −0.303 and −0.325, and *P*<0.0001, respectively) and LAIR1 on NK and NK^dim^ cells (r=−0.209 and −0.220, and *P*=0.002 and 0.0012, respectively) in the Spearman correlation analysis. However, it was not correlated with NKG2A on NK, NK^bright^, and NK^dim^ cells (*P*>0.05) (Figure 4A–L). Serum HBV pgRNA was not only positively associated with PD1 on NK^bright^ cells in both the univariate and multivariate linear regression analyses (B=0.030 and 0.001; both *P*<0.01), but also positively associated with NKG2A and Tim3 on NK^bright^ cells in multivariate linear regression analysis (B=0.116 and 0.048; *P*=0.001 and 0.020, respectively). It was also negatively associated with LAIR1 on NK and NK^dim^ cells in univariate linear regression analysis (B=−0.019 and −0.019; both *P*<0.05) (Supplementary Table S4). Thus, serum HBV pgRNA tended to be positively associated with inhibitory markers on NK^bright^ cells, but negatively associated with inhibitory markers on NK^dim^ cells.

Furthermore, regarding inhibitory markers, PD1 expression on NK cells and subsets was higher in the IA group compared with the IT, IC and GZ groups, while Tim3 expression was lowest in the IA group. No between-group differences in LAIR-1 or NKG2A on NK cell or subsets were found, except that NKG2A expression on NK^bright^ cells was higher in the IT group than the IA and IC groups using the K–W test and Bonferroni method analyses (Supplementary Figure S4).

### Associations with serum cytokines and chemokines

IL-7, IL-12, and PGE2 are critical molecules for the development and function of NK cells. Therefore, we explored the associations of serum HBV pgRNA with the serum levels of these cytokines (Supplementary Figure S5A–C). Serum HBV pgRNA was positively correlated with IL-7 and IL-12P40 (r=0.230 and 0.296, and *P*=0.0238 and 0.0032, respectively), which promote NK cell development and cytotoxicity. However, it was negatively correlated with PGE2 (r=−0.247 and *P*=0.0015), which inhibits NK cell function and activation. Additionally, the serum IL-7 level was highest in the IT group, the IL-12 level was higher in the IT and IA groups than the IC group and the PGE2 level was lower in the IA group than the IT and IC groups using the K–W test and Bonferroni method analyses (Supplementary Figure S5DF).

## Discussion

The present study explored the relationship between serum HBV pgRNA and NK cell immunity in CHB patients. We found that serum HBV pgRNA had varied relationships with different NK cell subsets. Although serum HBV pgRNA was negatively associated with NK and NK^dim^ cell frequency, it was positively associated with activation markers (NKp44 and NKp46) and negatively associated with inhibitory markers on these cells. In contrast, it was positively associated with NK^bright^ cell frequency and cytokine production of NK^bright^ cells but negatively associated with activation markers (NKp44 and NKp46) and positively associated with inhibitory markers on these cells. Moreover, it was positively associated with serum IL-7 and IL-12P40 (NK cell-promoting cytokines), but negatively associated with serum PGE2 (which negatively regulates NK cells).

NK cells in the peripheral blood are divided into two major subsets according to CD56 expression, referred to as CD56^dim^ (NK^dim^) cells and CD56^high^ (NK^bright^) cells. Most NK cells are NK^dim^, while <15% are NK^bright^ [17]. NK cells defend against virus infection and tumors, and the two subsets of NK cells play different roles in immunity. Upon stimulation by T cells, dendritic cells (DCs) and monocytes, NK^bright^ cells promptly produce high levels of chemokines and cytokines to regulate the immune response and act as a bridge between innate and adaptive immunity [18–20]. However, NK^bright^ cells do not readily express the intracellular cytotoxicity molecules granzymes A and B and perforin [21]. Therefore, they are thought to have poor cytotoxicity and more immune-modulatory activity. In contrast, NK^dim^ cells contain more cytotoxic granules and are more efficient regarding cytotoxicity and antibody-dependent cellular cytotoxicity (ADCC) [22–24]. In this study, serum HBV pgRNA had different relationships with NK, NK^dim^ and NK^bright^ cell parameters, which might be related to the different characteristics of these NK cell subsets.

Laroni et al. found that NK^bright^ cells could be induced to suppress CD4^+^ T-cell proliferation via the use of natural cytotoxicity receptors (NCRs) [25]. Morandi et al. found that NK^bright^ cells decreased T-cell proliferation by releasing adenosine [26]. NK^bright^ cells were also found to be cytotoxic toward T cells in daclizumab-treated patients [27]. T cells, especially virus-specific CD8^+^ T cells, play a crucial role in the antiviral response to HBV and pathogenesis of HBV infection. NK cells have been shown to have a negative effect on T cell immunity during chronic HBV infection [28–30]. Boni et al. observed an increase in NK^bright^ cells and a low level of HBV-specific T cells in patients with chronic HBV infection, and the authors proposed that NK^bright^ cells might promote HBV chronicity by killing T cells in a TNF-related apoptosis-inducing ligand (TRAIL)-dependent process. After antiviral therapy reduced HBV DNA and cleared HBsAg, HBV-specific T cells were no longer suppressed by NK cells [31]. Peppa et al. showed that activated NK cells highly expressed TRAIL and thereby hindered the function of HBV-specific T cells and promoted their apoptosis in HBV-infected inflammatory livers, preventing antiviral immunity in CHB [32]. Our previous study showed that Th1 immunity and cytotoxic lymphocytes (CTLs) were negatively correlated with the serum HBV pgRNA level [15]. In the current study, HBV pgRNA had a positive relationship with NK^bright^ cell frequency and cytokine production. Therefore, we speculate that HBV pgRNA might suppress Th1 cell antiviral immunity by enhancing NK^bright^ cell functions, which would contribute to HBV persistence in CHB patients. However, it is hard to explain why serum HBV pgRNA was positively associated with activation markers on NK and NK^dim^ cells. This needs further research.

NK cells recognize cells infected by viruses and clear the virus infection by producing cytokines [33]. NK cells contribute to HBV clearance [34] and their early activation can enhance HBV clearance in woodchuck models and individuals with acute HBV infection [35,36]. During acute HBV infection, antiviral cytokines mainly come from NK cells that accumulate in the liver [37]. However, during chronic HBV infection, NK cell immunity is gradually reduced, followed by enhancement of the adaptive immune response [9]. Zhang et al. found a lower level of circulating NK cells in CHB patients than HCs, especially during the IA phase of CHB, which might be due to the NK cells being more prone to apoptosis under the proinflammatory conditions during this phase, and HBV may significantly inhibit NK cell proliferation [38]. Tjwa et al. demonstrated that the NKp30^+^ NK cell frequency dramatically decreased in CHB patients, which was associated with the HBV level [8]. We observed that serum HBV pgRNA level, a new HBV marker, was negatively associated with NK and NK^dim^ cell frequency, suggesting that serum HBV pgRNA might also had negative association with NK cell proliferation.

Activation and inhibition of NK cells are strictly regulated to ensure full reactivity, appropriate immune monitoring, and self-tolerance, while avoiding excessive activity that can lead to inflammation or autoimmune diseases. A shift in the balance between activation and inhibition regulates NK cell responses. NK cells up-regulate inhibitory signals when interacting with peripheral tissues. When they come across target cells, the increase in activating receptor stimulation and/or the lack of inhibitory signals shifts the balance towards NK cell activation [39]. HBV infection can change the activation state and receptor expression pattern of NK cells. The up- or down-regulation of certain activation and inhibitory markers was reportedly correlated with the serum HBV DNA level [8]. Li et al. demonstrated that low HBV load and liver inflammation up-regulated NKp46 on NK cells in CHB, which facilitated inhibition of HBV replication and liver inflammation [40]. Alter et al. revealed that NKp30 and NKp46 down-regulation on NK cells was strongly related to HCV clearance [41], suggesting an indirect link between these natural cytotoxic receptors on NK cells and virus clearance. Similar to these previous results, we also found associations of serum HBV pgRNA with activation and inhibitory markers on NK cells. It was very interesting to observe that though the serum HBV pgRNA level was negatively associated with NK and NK^dim^ cell frequency, it was positively correlated with activation markers on these cells. Regarding NK^bright^ cells, the serum HBV pgRNA level was positively associated with NK^bright^ cell frequency and cytokine production, but negatively associated with activation markers and positively associated with inhibitory markers on the cells, indicating a complex association with NK^bright^ cells. All of these results demonstrated that the serum HBV pgRNA level had varied relationships with NK and subset cells, indicating that although HBV pgRNA might suppress NK and NK^dim^ cell proliferation, it may promote the activation of these cells, while it may promote both the proliferation of NK^bright^ cells and their function (in some respects, though it is negatively and positively associated with activation and inhibitory markers, respectively).

In mouse models, IL-7 is confirmed as an important cytokine for NK cell development in the thymus (with decreased NK cell generation during IL-7 production deficiency), but it does not affect NK cell development in the bone marrow [42]. Thymic NK cells regulate T and B cells in an IL7-dependent manner [43]. IL-12 induces NK cells to produce cytokines and enhances NK cell cytotoxicity, and it slightly increases the proliferation of resting peripheral blood NK cells [44]. In contrast, PGE2 inhibits NK cell function and activation and can promote antigenic immune escape via PGE2 receptors 2 and 4 (EP2 and EP4) on NK cells [45,46]. Bonavita et al. reported that PGE2 inhibited the early activation of NK cells and subsequently blocked T- and B-cell recruitment [47]. We also found that serum HBV pgRNA was associated with these molecules, which may partly explain how HBV pgRNA affects NK cell immunity.

In summary, we showed that the serum HBV pgRNA level had opposite associations with NK^dim^ and NK^bright^ cell frequency and function, suggesting that HBV pgRNA may play a complicated role in regulating NK cell-related immunity. Although the NK and NK^dim^ cell frequencies were suppressed, serum HBV pgRNA was still positively associated with NK^bright^ cells, which might limit T-cell responses, helping HBV to escape host immunity. However, the study is an observational study, and further experiments are still required to understand the molecular mechanism behind these findings. Understanding the molecular mechanism by which HBV evades host immune responses might help to develop new immunotherapeutic strategies to cure chronic HBV infection.

## Supplementary Material

Supplementary Figures S1-S5 and Tables S1-S4

## Data Availability

The data presented in the present study are available on request from the corresponding author.

## Abbreviations

ALB: albumin
ALT: alanine aminotransferase
APC: allophycocyanin
AST: aspartate aminotransferase
cccDNA: covalently closed circular DNA
CHB: chronic hepatitis B
FITC: fluorescein isothiocyanate
GLB: globulin
GZ: gray zone
HBeAb: HBV e antibody
HBeAg: HBV e antigen
HBsAb: HBV surface antibody
HBsAg: HBV surface antigen
HBV: hepatitis B virus
HC: healthy control
HCV: hepatitis C virus
IA: immune-active
IC: inactive carrier
IFN: interferon
IL: interleukin
IT: immune-tolerant
K–W: Kruskal–Wallis
LAIR1: leukocyte-associated immunoglobulin-like receptor 1
NK: natural killer
PBMC: peripheral blood mononuclear cell
PD1: programmed cell death protein 1
PE: phycoerythrin
PGE2: prostaglandin E2
pgRNA: pregenomic RNA
qPCR: quantitative polymerase chain reaction
TBIL: total bilirubin
Tim3: T cell immunoglobulin and mucin domain-containing protein 3
TNF: tumor necrosis factor
TRAIL: TNF-related apoptosis-inducing ligand

## References

[1] Lavanchy D. (2005) Worldwide epidemiology of HBV infection, disease burden, and vaccine prevention. J. Clin. Virol. 34, S1–S3 10.1016/S1386-6532(05)00384-7 16461208

[2] Guidotti L.G. and Chisari F.V. (2006) Immunobiology and pathogenesis of viral hepatitis. Annu. Rev. Pathol. 1, 23–61 10.1146/annurev.pathol.1.110304.100230 18039107

[3] O’Brien K.L. and Finlay D.K. (2019) Immunometabolism and natural killer cell responses. Nat. Rev. Immunol. 19, 282–290 10.1038/s41577-019-0139-2 30808985

[4] Katchar K., Drouin E. and Steere A. (2013) Natural killer cells and natural killer T cells in Lyme arthritis. Arthritis Res. Therapy 15, R183 10.1186/ar4373 PMC3978756 24286535

[5] Guidotti L.G., Rochford R., Chung J., Shapiro M., Purcell R. and Chisari F.V. (1999) Viral clearance without destruction of infected cells during acute HBV infection. Science 284, 825–829 10.1126/science.284.5415.825 10221919

[6] Schuch A., Hoh A. and Thimme R. (2014) The role of natural killer cells and CD8(+) T cells in hepatitis B virus infection. Front. Immunol. 5, 258 10.3389/fimmu.2014.00258 24917866 PMC4042360

[7] Fisicaro P., Valdatta C., Boni C., Massari M., Mori C., Zerbini A. et al. (2009) Early kinetics of innate and adaptive immune responses during hepatitis B virus infection. Gut 58, 974–982 10.1136/gut.2008.163600 19201769

[8] Tjwa E.T., van Oord G.W., Hegmans J.P., Janssen H.L. and Woltman A.M. (2011) Viral load reduction improves activation and function of natural killer cells in patients with chronic hepatitis B. J. Hepatol. 54, 209–218 10.1016/j.jhep.2010.07.009 21095036

[9] Li Y., Wang J.-J., Gao S., Liu Q., Bai J., Zhao X.-Q. et al. (2014) Decreased peripheral natural killer cells activity in the immune activated stage of chronic hepatitis B. PLoS ONE 9, e86927 10.1371/journal.pone.0086927 24520324 PMC3919705

[10] Oliviero B., Varchetta S., Paudice E., Michelone G., Zaramella M., Mavilio D. et al. (2009) Natural killer cell functional dichotomy in chronic hepatitis B and chronic hepatitis C virus infections. Gastroenterology 137, 1151–1160, 1160.e1-e7, 10.1053/j.gastro.2009.05.047 19470388

[11] Li X., Zhou L., Gu L., Gu Y., Chen L., Lian Y. et al. (2017) Veritable antiviral capacity of natural killer cells in chronic HBV infection: an argument for an earlier anti-virus treatment. J. Transl. Med. 15, 220 10.1186/s12967-017-1318-1 29089040 PMC5663047

[12] Gill U.S., Peppa D., Micco L., Singh H.D., Carey I., Foster G.R. et al. (2016) Interferon alpha induces sustained changes in NK cell responsiveness to hepatitis B viral load suppression in vivo. PLoS Pathog. 12, e1005788–e 10.1371/journal.ppat.1005788 27487232 PMC4972354

[13] Yang Y., Han Q., Zhang C., Xiao M. and Zhang J. (2016) Hepatitis B virus antigens impair NK cell function. Int. Immunopharmacol. 38, 291–297 10.1016/j.intimp.2016.06.015 27341035

[14] Zimmer C.L., Rinker F., Höner zu Siederdissen C., Manns M.P., Wedemeyer H., Cornberg M. et al. (2018) Increased NK cell function after cessation of long-term nucleos(t)ide analogue treatment in chronic hepatitis B is associated with liver damage and HBsAg loss. J. Infect. Dis. 217, 1656–1666 10.1093/infdis/jiy097 29471497

[15] Gu Y., Chen L., Lian Y., Gu L., Chen Y., Bi Y. et al. (2020) Serum HBV pregenomic RNA is correlated with Th1/Th2 immunity in treatment-naive chronic hepatitis B patients. J. Med. Virol. 92, 317–328 10.1002/jmv.25612 31642539 PMC7004183

[16] Terrault N.A., Lok A.S.F., McMahon B.J., Chang K.M., Hwang J.P., Jonas M.M. et al. (2018) Update on prevention, diagnosis, and treatment of chronic hepatitis B: AASLD 2018 hepatitis B guidance. Hepatology 67, 1560–1599 10.1002/hep.29800 29405329 PMC5975958

[17] Lanier L., Le A., Phillips J., Warner N. and Babcock G. (1983) Subpopulations of human natural killer cells defined by expression of the Leu-7 (HNK-1) and Leu-11 (NK-15) antigens. J. Immunol. 131, 1789–1796 6225799

[18] Cooper M., Fehniger T., Ponnappan A., Mehta V., Wewers M. and Caligiuri M. (2001) Interleukin-1beta costimulates interferon-gamma production by human natural killer cells. Eur. J. Immunol. 31, 792–801 10.1002/1521-4141(200103)31:3<792::AID-IMMU792>3.0.CO;2-U 11241284

[19] Fehniger T., Shah M., Turner M., VanDeusen J., Whitman S., Cooper M. et al. (1999) Differential cytokine and chemokine gene expression by human NK cells following activation with IL-18 or IL-15 in combination with IL-12: implications for the innate immune response. J. Immunol. 162, 4511–4520 10201989

[20] Fehniger T., Cooper M., Nuovo G., Cella M., Facchetti F., Colonna M. et al. (2003) CD56bright natural killer cells are present in human lymph nodes and are activated by T cell-derived IL-2: a potential new link between adaptive and innate immunity. Blood 101, 3052–3057 10.1182/blood-2002-09-2876 12480696

[21] Michel T., Poli A., Cuapio A., Briquemont B., Iserentant G., Ollert M. et al. (2016) Human CD56bright NK cells: an update. J. Immunol. 196, 2923–2931 10.4049/jimmunol.1502570 26994304

[22] Cichicki F., Schlums H., Theorell J., Tesi B., Miller J., Ljunggren H. et al. (2016) Diversification and functional specialization of human NK cell subsets. Curr. Top. Microbiol. Immunol. 395, 63–94 26472216 10.1007/82_2015_487

[23] Cooper M., Fehniger T. and Caligiuri M. (2001) The biology of human natural killer-cell subsets. Trends Immunol. 22, 633–640 10.1016/S1471-4906(01)02060-9 11698225

[24] Freud A., Mundy-Bosse B., Yu J. and Caligiuri M. (2017) The broad spectrum of human natural killer cell diversity. Immunity 47, 820–833 10.1016/j.immuni.2017.10.008 29166586 PMC5728700

[25] Laroni A., Armentani E., Kerlero de Rosbo N., Ivaldi F., Marcenaro E., Sivori S. et al. (2016) Dysregulation of regulatory CD56(bright) NK cells/T cells interactions in multiple sclerosis. J. Autoimmun. 72, 8–18 10.1016/j.jaut.2016.04.003 27157273

[26] Morandi F., Horenstein A., Chillemi A., Quarona V., Chiesa S., Imperatori A. et al. (2015) CD56brightCD16- NK cells produce adenosine through a CD38-mediated pathway and act as regulatory cells inhibiting autologous CD4^+^ T cell proliferation. J. Immunol. 195, 965–972 10.4049/jimmunol.1500591 26091716

[27] Bielekova B., Catalfamo M., Reichert-Scrivner S., Packer A., Cerna M., Waldmann T. et al. (2006) Regulatory CD56(bright) natural killer cells mediate immunomodulatory effects of IL-2Ralpha-targeted therapy (daclizumab) in multiple sclerosis. Proc. Natl. Acad. Sci. U.S.A. 103, 5941–5946 10.1073/pnas.0601335103 16585503 PMC1458677

[28] Jiang S., Zhu Y., Cheng C., Li Y., Ma T., Peng Z. et al. (2020) NK cells contribute to hepatic CD8 T cell failure in hepatitis B virus-carrier mice after alcohol consumption. Virus Res. 286, 198085 10.1016/j.virusres.2020.198085 32622853

[29] Li H., Zhai N., Wang Z., Song H., Yang Y., Cui A. et al. (2018) Regulatory NK cells mediated between immunosuppressive monocytes and dysfunctional T cells in chronic HBV infection. Gut 67, 2035–2044 10.1136/gutjnl-2017-314098 28899983 PMC6176520

[30] Peppa D., Gill U., Reynolds G., Easom N., Pallett L., Schurich A. et al. (2013) Up-regulation of a death receptor renders antiviral T cells susceptible to NK cell-mediated deletion. J. Exp. Med. 210, 99–114 10.1084/jem.20121172 23254287 PMC3549717

[31] Boni C., Lampertico P., Talamona L., Giuberti T., Invernizzi F., Barili V. et al. (2015) Natural killer cell phenotype modulation and natural killer/T-cell interplay in nucleos(t)ide analogue-treated hepatitis e antigen-negative patients with chronic hepatitis B. Hepatology 62, 1697–1709 10.1002/hep.28155 26361374

[32] Peppa D., Gill U.S., Reynolds G., Easom N.J., Pallett L.J., Schurich A. et al. (2013) Up-regulation of a death receptor renders antiviral T cells susceptible to NK cell-mediated deletion. J. Exp. Med. 210, 99–114 10.1084/jem.20121172 23254287 PMC3549717

[33] Maini M.K. and Gehring A.J. (2016) The role of innate immunity in the immunopathology and treatment of HBV infection. J. Hepatol. 64, S60–S70 10.1016/j.jhep.2016.01.028 27084038

[34] Yang P.L., Althage A., Chung J. and Chisari F.V. (2002) Hydrodynamic injection of viral DNA: a mouse model of acute hepatitis B virus infection. Proc. Natl. Acad. Sci. U.S.A. 99, 13825–13830 10.1073/pnas.202398599 12374864 PMC129782

[35] Guy C.S., Mulrooney-Cousins P.M., Churchill N.D. and Michalak T.I. (2008) Intrahepatic expression of genes affiliated with innate and adaptive immune responses immediately after invasion and during acute infection with woodchuck hepadnavirus. J. Virol. 82, 8579–8591 10.1128/JVI.01022-08 18596101 PMC2519695

[36] Webster G.J.M., Reignat S., Maini M.K., Whalley S.A., Ogg G.S., King A. et al. (2000) Incubation phase of acute hepatitis B in man: dynamic of cellular immune mechanisms. Hepatology 32, 1117–1124 10.1053/jhep.2000.19324 11050064

[37] Jo J., Tan A.T., Ussher J.E., Sandalova E., Tang X.-Z., Tan-Garcia A. et al. (2014) Toll-like receptor 8 agonist and bacteria trigger potent activation of innate immune cells in human liver. PLoS Pathog. 10, e1004210 10.1371/journal.ppat.1004210 24967632 PMC4072808

[38] Zhang Q.F., Shao J.Y., Yin W.W., Xia Y., Chen L., Wang X. et al. (2016) Altered immune profiles of natural killer cells in chronic hepatitis B patients: a systematic review and meta-analysis. PLoS ONE 11, e0160171 10.1371/journal.pone.0160171 27513564 PMC4981347

[39] Jelencic V., Sestan M., Kavazovic I., Lenartic M., Marinovic S., Holmes T.D. et al. (2018) NK cell receptor NKG2D sets activation threshold for the NCR1 receptor early in NK cell development. Nat. Immunol. 19, 1083–1092 10.1038/s41590-018-0209-9 30224819 PMC6166863

[40] Li W., Jiang Y., Wang X., Jin J., Qi Y., Chi X. et al. (2015) Natural killer p46 controls hepatitis B virus replication and modulates liver inflammation. PloS ONE 10, e0135874 10.1371/journal.pone.0135874 26291078 PMC4546267

[41] Alter G., Jost S., Rihn S., Reyor L.L., Nolan B.E., Ghebremichael M. et al. (2011) Reduced frequencies of NKp30+NKp46+, CD161+, and NKG2D+ NK cells in acute HCV infection may predict viral clearance. J. Hepatol. 55, 278–288 10.1016/j.jhep.2010.11.030 21168454 PMC3729214

[42] Vosshenrich C., Ranson T., Samson S., Corcuff E., Colucci F., Rosmaraki E. et al. (2005) Roles for common cytokine receptor gamma-chain-dependent cytokines in the generation, differentiation, and maturation of NK cell precursors and peripheral NK cells in vivo. J. Immunol. 174, 1213–1221 10.4049/jimmunol.174.3.1213 15661875

[43] Ma A., Koka R. and Burkett P. (2006) Diverse functions of IL-2, IL-15, and IL-7 in lymphoid homeostasis. Annu. Rev. Immunol. 24, 657–679 10.1146/annurev.immunol.24.021605.090727 16551262

[44] Trinchieri G. (1994) Interleukin-12: a cytokine produced by antigen-presenting cells with immunoregulatory functions in the generation of T-helper cells type 1 and cytotoxic lymphocytes. Blood 84, 4008–4027 10.1182/blood.V84.12.4008.bloodjournal84124008 7994020

[45] Harizi H. (2013) Reciprocal crosstalk between dendritic cells and natural killer cells under the effects of PGE2 in immunity and immunopathology. Cell Mol. Immunol. 10, 213–221 10.1038/cmi.2013.1 23524652 PMC4012770

[46] Bonavita E., Bromley C., Jonsson G., Pelly V., Sahoo S., Walwyn-Brown K. et al. (2020) Antagonistic inflammatory phenotypes dictate tumor fate and response to immune checkpoint blockade. Immunity 53, 1215.e8–1229.e8 10.1016/j.immuni.2020.10.020 33220234 PMC7772804

[47] Knudsen N. and Manguso R. (2020) Tumor-derived PGE2 gives NK cells a headache. Immunity 53, 1131–1132 10.1016/j.immuni.2020.11.018 33326763

