# Supplementary material for: Serum HBV pregenomic RNA exhibited opposite associations with NKdim and NKbright cell immunity in treatment-naïve chronic hepatitis B patients
Source: Biosci Rep. 2021 Jul 2;41(7):BSR20210600. doi: 10.1042/BSR20210600 (PMC8255538; doi:10.1042/BSR20210600)
Supplement: Supplementary Figures S1-S5 and Tables S1-S4 [file BSR-2021-0600_supp.pdf]

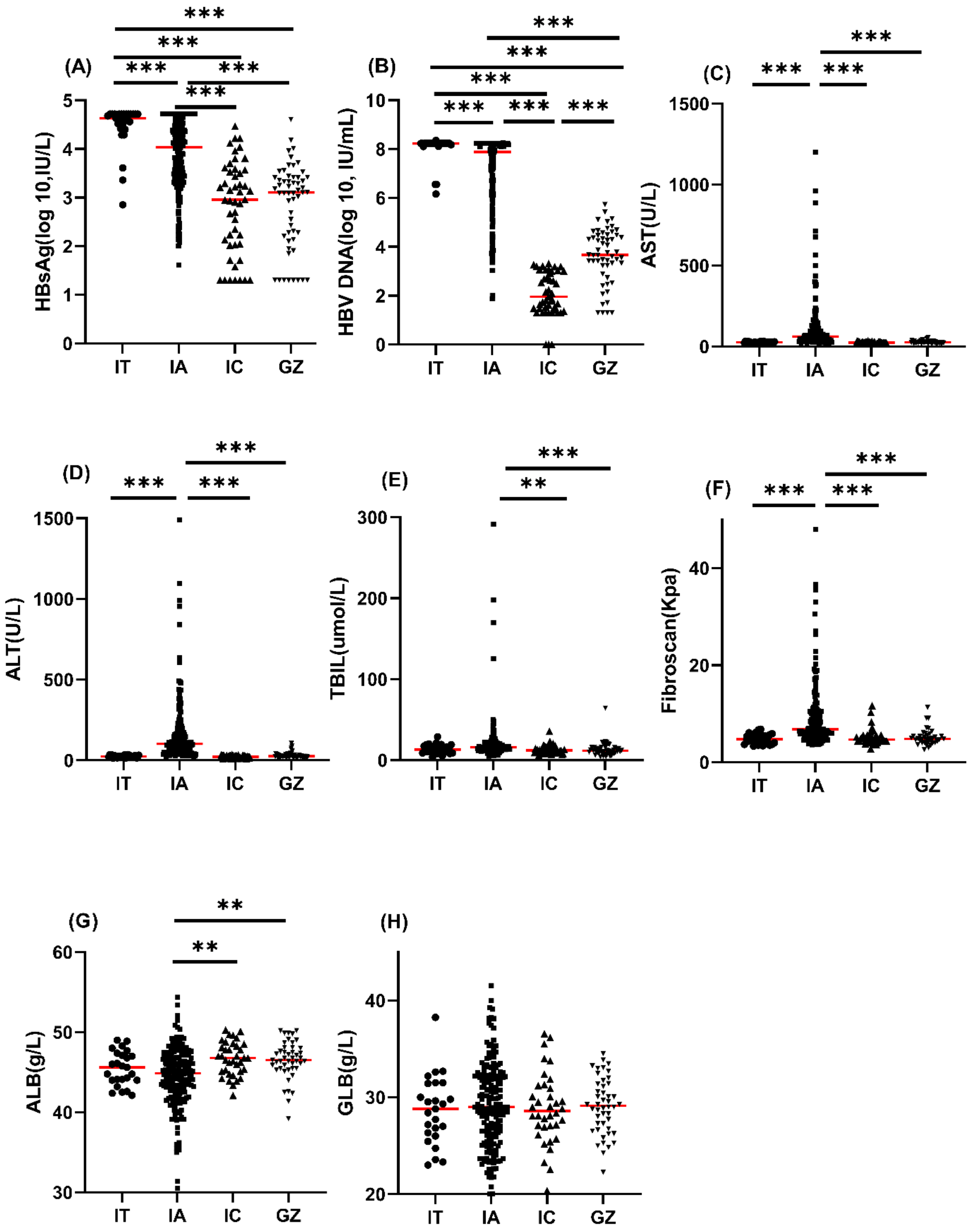

Supplementary Figure 1. **Liver inflammation, fibrosis, virological characteristics significantly differed among groups.**
HBsAg（A）, HBV DNA(B), AST(C), ALT(D), TBIL(E), FibroScan(F), ALB(G) and GLB(H)levels significantly differed among groups.

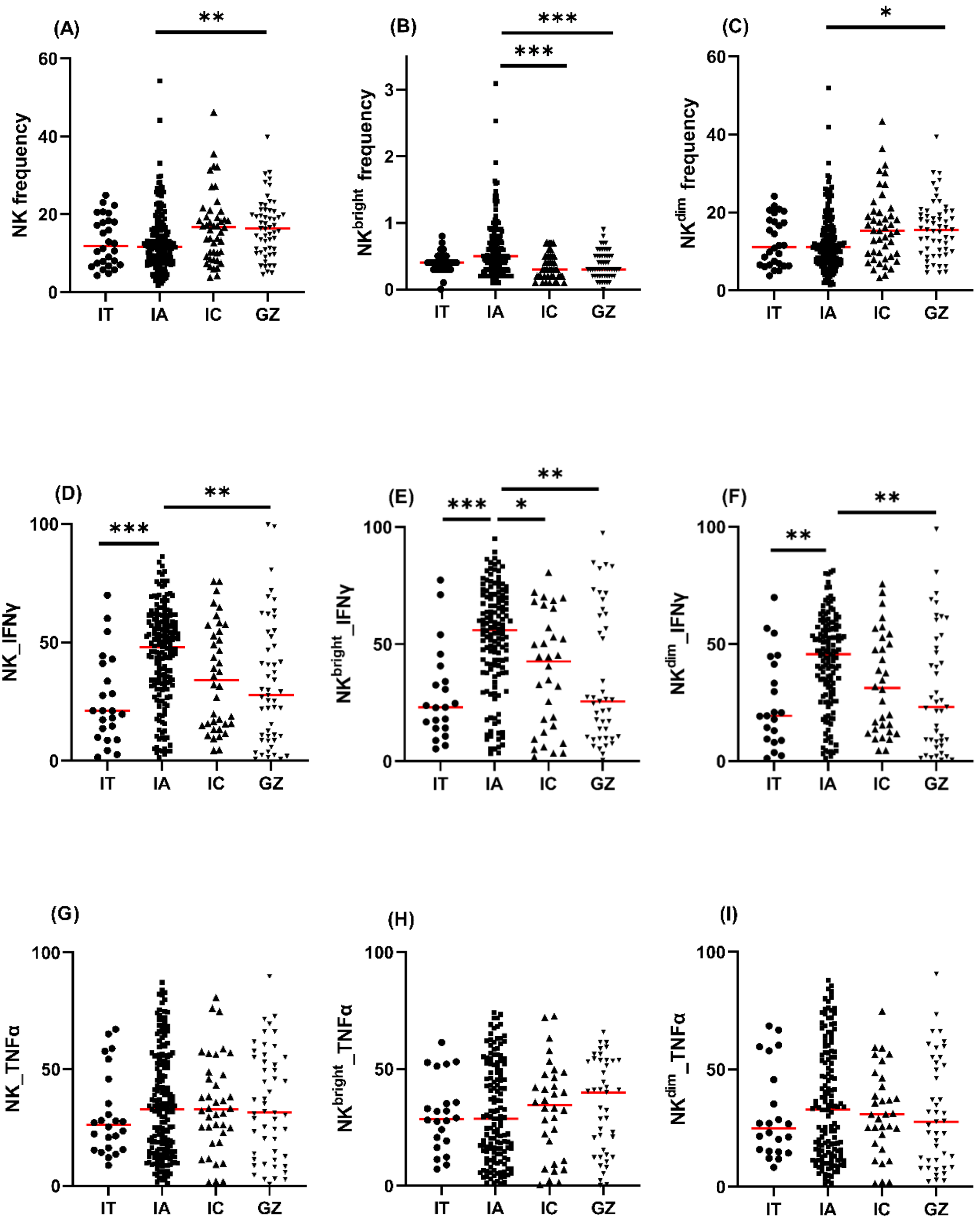

Supplementary Figure 2. **NK cell and subset frequencies and antiviral cytokine (IFN-γ and TNF-α) production significantly differed among groups.**
NK cell and subset frequencies(A-C) and IFN-γ (D-F) and TNF-α(G-I) production significantly differed among groups.

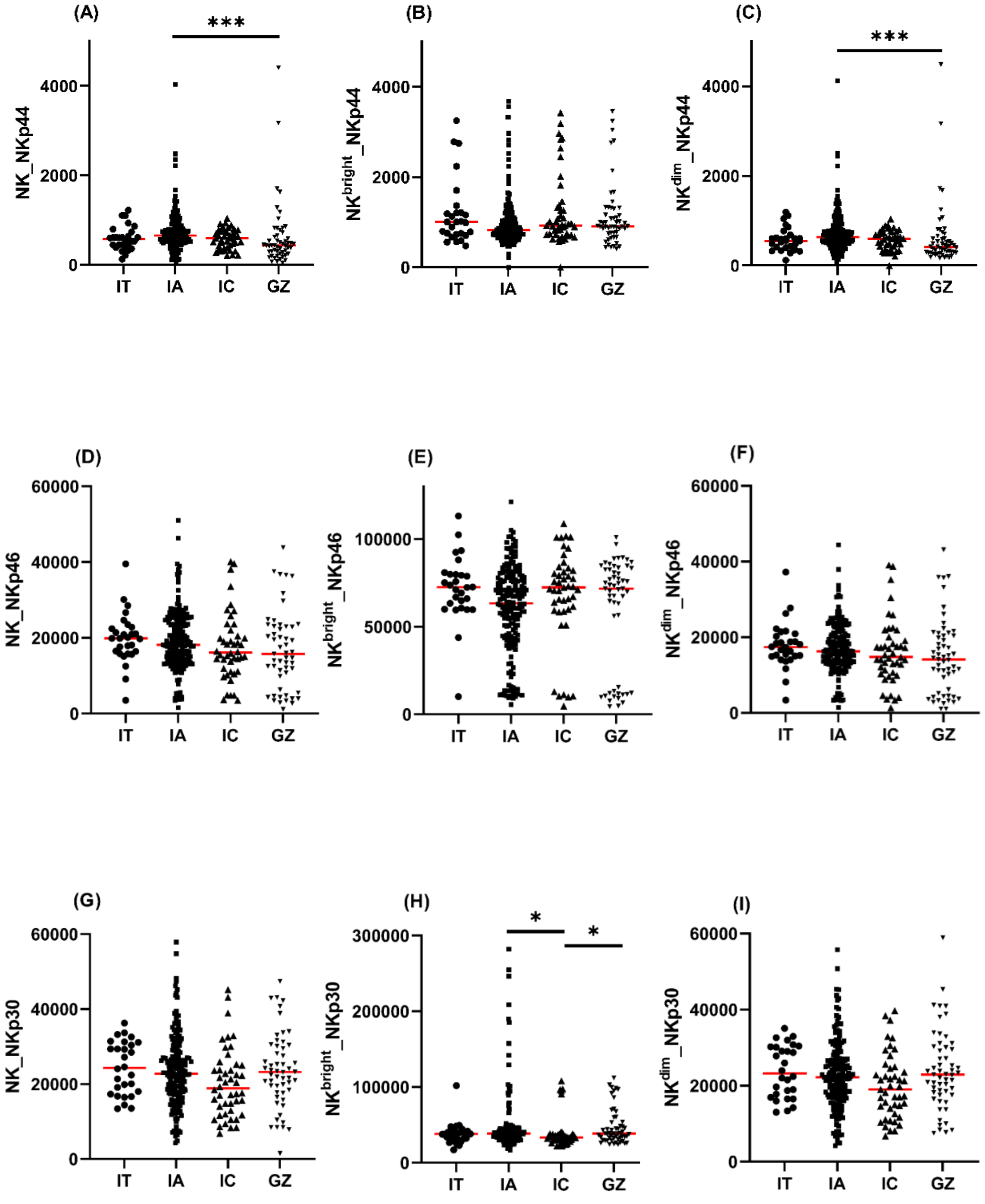

Supplementary Figure 3. **Activation markers (NKP44, NKP46 and NKp30) on NK cells and subsets significantly differed among groups.**

NKP44(A-C), NKP46(D-F) and NKp30(G-I) on NK cells and subsets significantly differed among groups.

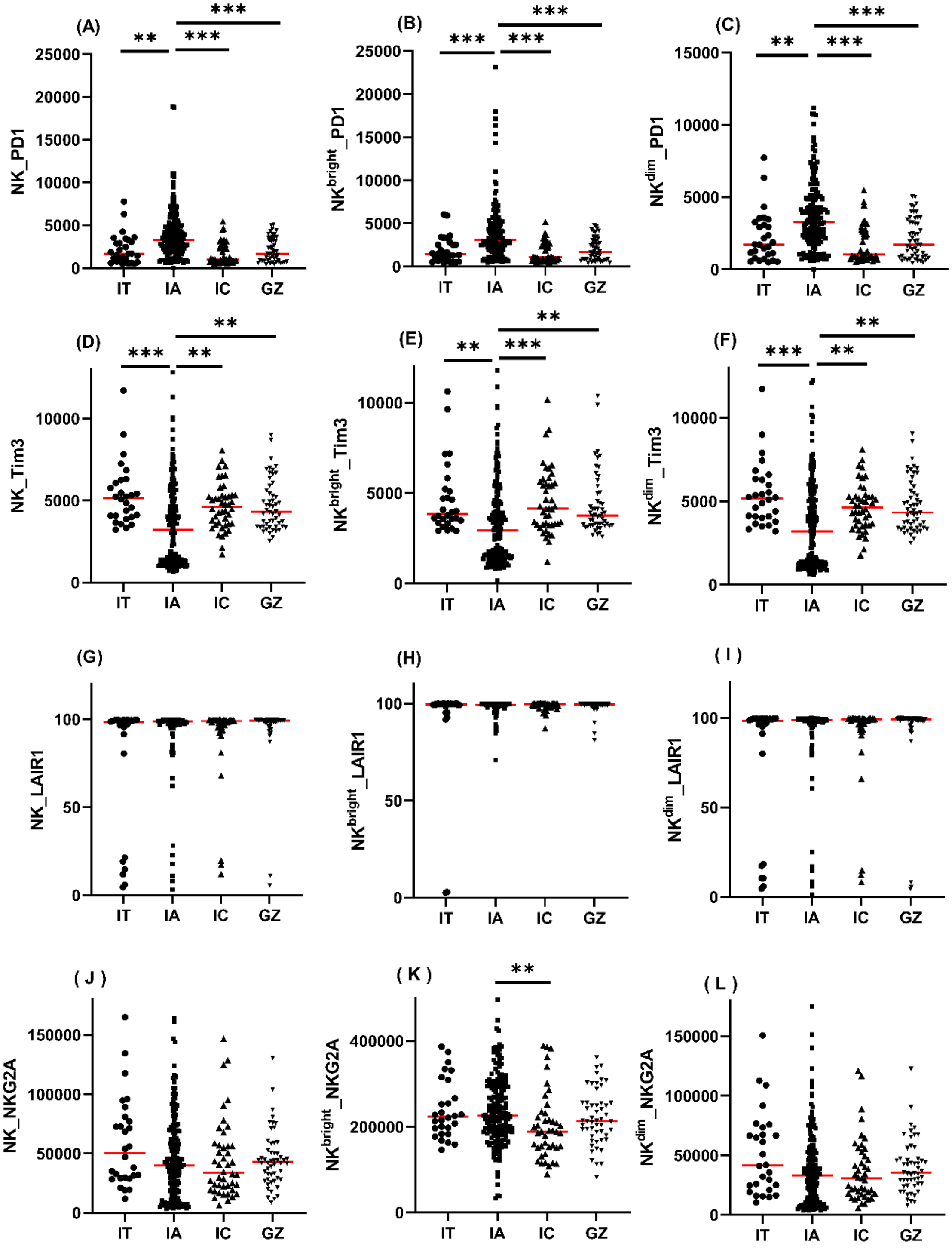

Supplementary Figure 4. Inhibitory markers (PD1, Tim3, LAIR1 and NKG2A) on NK cells and subsets significantly differed among groups.

PD1(A-C), Tim3(D-F), LAIR1(G-I) and NKG2A(J-L) on NK cells and subsets significantly differed among groups.

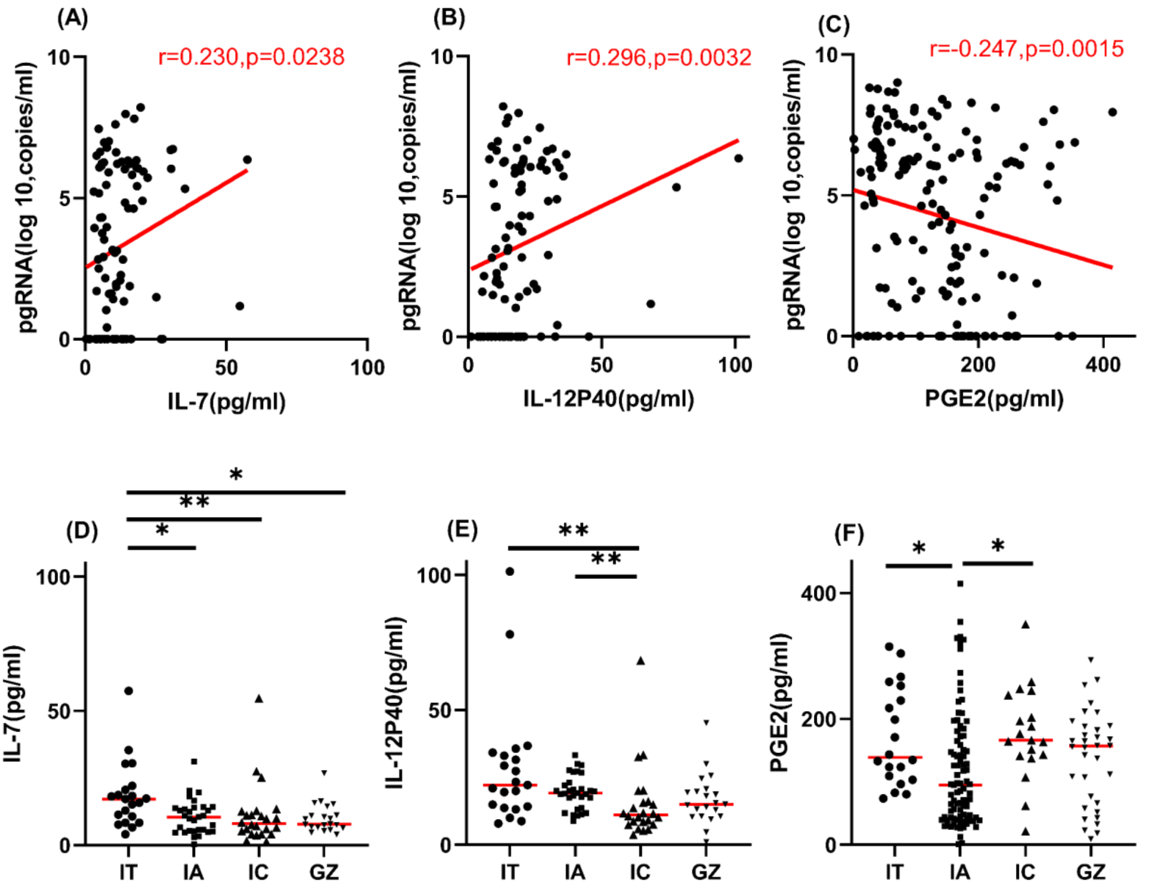

Supplementary Figure 5. Correlations of serum HBV pgRNA with NK cell-related serum cytokines (IL-7, IL-12P40 and PGE2) and differences of NK cell-related serum cytokines among groups.

(A-C) Correlations of serum HBV pgRNA with NK cell-related serum cytokines (IL-7, IL-12P40 and PGE2). (D-F) NK cell-related serum cytokines (IL-7, IL-12P40, and PGE2) significantly differed among groups.

Supplementary Table 1. Univariate and multivariate linear regression analysis of factors of HBV virological Characteristics , liver inflammation and fibrosis associated with serum pgRNA

	Univariate			Multivariate		
	**B**	**95% CI**	**P value**	**B**	**95% CI**	**P value**
Log HBV DNA	0.903	0.810, 0.980	***0.000***	0.564	0.424, 0.705	***0.000***
HBsAb（positive）	0.791	-0.331, 1.913	0.167	0.043	-0.263, 1.152	0.232
HBcAb	-3.731	-19.104, 10.561	0.624	-0.065	-17.827, 0.863	0.070
Log HBsAg	1.845	1.543, 2.092	***0.000***	0.094	0.043, 0.662	0.059
HBeAb	0.367	0.291, 0.439	***0.000***	-0.033	-0.103, 0.038	0.483
HBeAg（positive）	4.341	3.885, 4.796	***0.000***	2.097	1.399, 2.795	***0.000***
AST	0.007	0.004, 0.009	***0.000***	-0.045	-0.008, 0.005	0.702
ALT	0.006	0.005, 0.008	***0.000***	0.005	0.002, 0.007	***0.000***
TBIL	0.019	0.005, 0.033	***0.008***	-0.041	-0.035, 0.019	0.552
Fibroscan	0.155	0.102, 0.209	***0.000***	0.089	0.028, 0.149	***0.004***
ALB	-0.219	-0.315, -0.122	***0.000***	-0.124	-0.521, 0.087	0.065
GLB	-0.005	-0.090, 0.081	0.915	-0.011	-0.291, 0.596	0.859

Abbreviations: pgRNA, pregenomic RNA ; CI, confidence interval; HBV, hepatitis B virus; HBsAg, hepatitis B surface antigen; HBcAb: hepatitis B core antibody ;HBeAb, antibody to HBV e antigen; HBeAg, HBV e antigen; HBsAb: antibody to hepatitis B surface antigen; AST, aspartate transaminase； ALT, alanine aminotransferase ; TBIL, total bilirubin; ALB, albumin; GLB, globulin.

Supplementary Table 2 .Univariate and multivariate linear regression analysis of NK cell frequency and antiviral cytokines of NK cells and tis subsets associated with serum pgRNA

	Univariate			Multivariate		
	**B**	**95% CI**	**P value**	**B**	**95% CI**	**P value**
NK	-0.073	-0.114, -0.032	***0.001***	-0.658	-1.219, -0.096	***0.022***
NK^{bright}	2.489	1.617, 3.362	***0.000***	2.885	1.920, 3.850	***0.000***
NK^{dim}	-0.076	-0.118, -0.035	***0.000***	0.602	0.032, 1.173	***0.039***
NK_IFN-γ	0.025	0.010, 0.040	***0.001***	-0.018	-0.114, 0.266	0.895
Nk^{bright}_IFN-γ	0.025	0.011, 0.039	***0.001***	0.035	0.020, 0.051	***0.000***
NK^{dim}_IFN-γ	0.026	0.009, 0.042	***0.002***	-0.035	-0.249, 0.102	0.782
NK_TNF-α	-0.002	-0.017, 0.013	0.781	0.030	0.005, 0.055	***0.017***
Nk^{bright}_ TNF-α	-0.009	-0.027, 0.009	0.328	-0.055	-0.084, -0.026	***0.000***
NK^{dim}_TNF-α	0.005	-0.011, 0.021	0.551	-0.09	-0.186, 0.140	0.889

Abbreviations: CI, confidence interval ; pgRNA, pregenomic RNA; IFN-γ,interferon-γ; TNF-α,tumor necrosis factor-α ;

Supplementary Table 3. Univariate and multivariate linear regression analysis of NK cell activating markers associated with serum pgRNA

	**Univariate**			**Multivariate**		
	**B**	**95% CI**	**P value**	**B**	**95% CI**	**P value**
NK_NKP30	0.001	-0.002, 0.004	0.550	0.086	-0.004, 0.061	0.164
NK_NKP44	0.090	0.022, 0.157	***0.009***	1.561	0.705, 2.417	***0.000***
NK_NKP46	0.005	0.001, 0.008	***0.018***	0.023	0.008, 0.037	***0.002***
NK^{bright}_NKP30	0.001	0.000, 0.001	0.315	0.005	-0.007, 0.002	0.940
NK^{bright}_NKP44	-0.17	-0.042, 0.008	0.193	-0.045	-0.074, -0.015	***0.003***
NK^{bright}_NKP46	-0.001	-0.002, 0.001	0.324	-0.003	-0.005, -0.001	***0.000***
NK^{dim}_NKP30	0.00018	-0.003, 0.004	0.924	0.099	-0.059, 0.007	0.103
NK^{dim}_NKP44	0.001	0.000, 0.001	***0.010***	-1.425	-2.260, -0.589	***0.001***
NK^{dim}_NKP46	0.004	-0.001, 0.008	0.091	-0.021	-0.038, -0.005	***0.012***

Abbreviations: CI, confidence interval ; pgRNA, pregenomic RNA;

Supplementary Table 4. Univariate and multivariate linear regression analysis of NK cell inhibitory markers associated with serum pgRNA

	**Univariate**			**Multivariate**		
	**B**	**95% CI**	**P value**	**B**	**95% CI**	**P value**
NK NKG2A	0.006	-0.015, 0.027	0.574	0. 0136	-0.115, 0.143	0.839
Nk^{bright} NKG2A	-0.004	-0.041, 0.032	0.825	0.116	0.045, 0.186	***0.001***
Nk^{dim} NKG2A	-0.001	-0.023, 0.021	0.946	0.001	-0.127, 0.126	0.995
NK_PD1	0.033	0.021, 0.045	***0.000***	−0.010	-0.027, 0.006	0.212
NK^{bright}_PD1	0.030	0.018, 0.041	***0.000***	0.001	0.001, 0.002	***0.009***
NK^{dim}_PD1	0.035	0.023, 0.048	***0.000***	0.009	-0.006, 0. 025	0.240
NK_Tim3	-0.008	-0.027, 0.011	0.425	-0.110	-0.330, 0.3025	0.324
NK^{bright}_Tim3	0.004	-0.015, -0.023	0.690	0.048	0.008, 0.110	***0.020***
NK^{dim}_Tim3	-0.007	-0.026, 0.013	0.503	0.056	-0.157, 0.088	0.607
NK_LAIR1	-0.019	-0.035, -0.004	***0.016***	0.049	-0.299, 0.397	0.782
Nk^{bright}_LAIR1	-0.032	-0.071, 0.007	0.105	-0.029	-0.076, 0.019	0.236
NK^{dim}_LAIR1	-0.019	-0.034, -0.004	***0.015***	-0.058	-0.389, -0.273	0.731

Abbreviations: CI, confidence interval ; pgRNA, pregenomic RNA;
